# Acute Trigger Finger Presenting as an Extensor Lag

**Published:** 2018-01-05

**Authors:** Stephen R. Ali, Hussein Mohamedbahi

**Affiliations:** Department of Plastic, Reconstructive and Burns Surgery, North Bristol NHS Trust, Bristol, United Kingdom

**Keywords:** stenosing tenosynovitis, trigger finger, extensor lag, steroid injection, management

**Figure:**
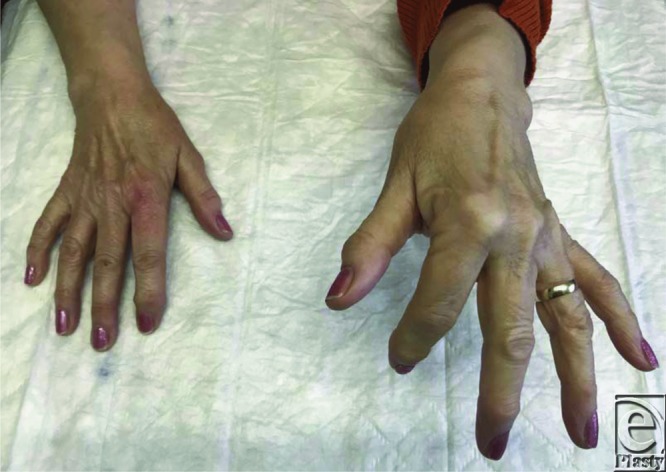


## DESCRIPTION

A 75-year-old right-hand–dominant woman presented with pain and swelling of the left index finger and was unable to straighten the digit after forcibly turning the garden tap on. There was no history of triggering. On examination, there was a palpable nodule proximal to the A1 pulley and no active or passive extension at the metacarpophalangeal (MCP) joint. There was full extension at the proximal interphalangeal (PIP) and distal interphalangeal (DIP) joints. X-ray study was unremarkable. She was managed with triamcinolone injection, which she responded to.

## QUESTIONS

1. What is the anatomy of the digital pulley system?
2. What are the differential diagnoses of an extensor lag to a digit?
3. How are trigger fingers assessed and classified?
4. What non-surgical and surgical treatment options exist for a trigger finger?

## DISCUSSION

**Figure 1:**
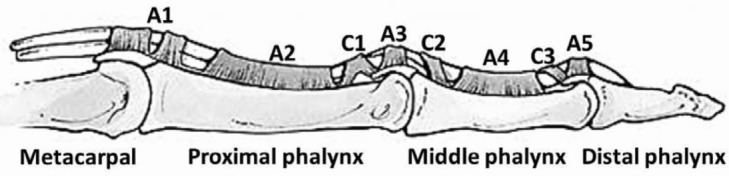
Anatomy of the digital pulley system.

The flexor sheath is a closed, double-walled, hollow, synovial-lined, fibro-osseous tunnel that is reinforced in sections that act as a pulley mechanism for the gliding of the flexor tendons within this narrow space.^1^ This pulley system is divided into 5 annular and 3 cruciate pulleys. The role of the annular pulleys is to prevent bowstringing during flexion, whereas the cruciate pulleys prevent sheath collapse and expansion during digital motion. The A1, A3, and A5 pulleys overlie the MCP, PIP, and DIP joints, respectively (Fig 1), and the A2 and A4 pulleys overlie the proximal aspect of the proximal phalanx and the middle aspect of the middle phalanx, respectively (remembered “proximal of the proximal” and “middle of the middle”). The A2 and A4 pulleys are biomechanically the most important pulleys and prevent bowstringing. The C1, C2, and C3 pulleys are interposed between the A2-A3, A3-A4, and A4-A5 pulleys at the level of the PIP and DIP joints.

**Figure 2:**
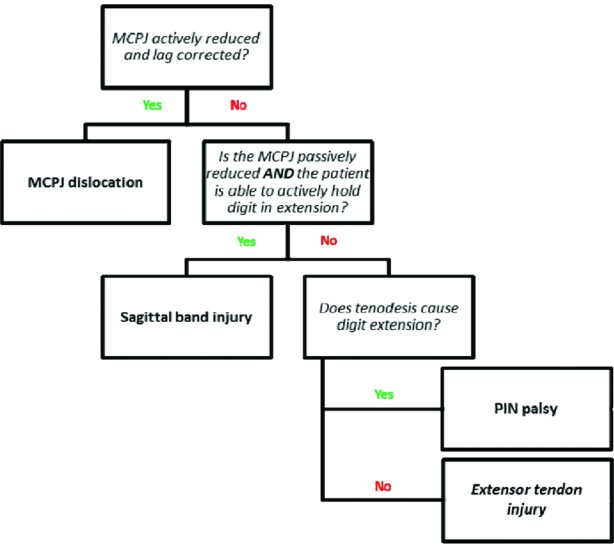
Flow diagram demonstrating the approach to extensor lag diagnosis. MCPJ indicates metacarpophalangeal joint; PIN, posterior interosseous nerve.

The most common differential diagnoses to consider in patient presenting with an acute extensor lag include extensor tendon injury (traumatic or attritional), sagittal band injury, MCP joint dislocation, or posterior interosseous nerve palsy.^2^^-^4 A considered history and careful examination will lead to the diagnosis with the described approach (Fig 2).

Trigger finger refers to mechanical impingement of a tendon caused by narrowing of its sheath.^5^ Mismatch occurs between the size of the flexor tendon sheath and its contents within the A1 pulley. Repeated attempts of the tendon to glide through the stenotic sheath lead to inflammation and resistance.^6^ This causes painful catching of the flexor tendon during flexion and extension in the chronic care setting.^5^ Occasionally, the digit will lock in flexion and require passive manipulation into extension. Rarely, a trigger finger presents acutely and may not actively or passively extend as in this case. The degree of triggering can be classified symptomatically and functionally in the chronic setting (Table 1).^5^

Corticosteroid injection is indicted for the primary trigger finger and the thumb and has a high rate of satisfaction for nondiabetic patients, single-digit involvement, a discrete palpable nodule, and a short history. Splinting can be considered for those who decline injection. Open surgical release is indicated for recalcitrant digits that have failed 1 or 2 corticosteroid injections.^7^ It is performed through a 1-cm longitudinal or oblique incision placed in the skin lines and directly over the A1 pulley.^5^ Percutaneous techniques have evolved as a safe alternative to open techniques with good medium-term outcomes, with reported success rates ranging between 74% and 94%.^5^ However, it may not be suitable for grade V disease with anecdotal reports of a high incomplete relief of triggering.^5^

## Figures and Tables

**Table 1:** Classification on the symptomatic severity of triggering

Grade I	Pain; history of catching but not demonstrable on physical examination; tenderness over the A1 pulley
Grade II	Demonstrable catching, but active extension of the digit
Grade III	Demonstrable catching requiring passive extension (grade IIIA) or inability to actively flex (grade IIIB)
Grade IV	Demonstrable catching with a fixed flexion contracture of the proximal interphalangeal joint
